# HIV status and survival of patients with pulmonary hypertension due to left heart disease: the Pan African Pulmonary Hypertension Cohort

**DOI:** 10.1038/s41598-023-36375-y

**Published:** 2023-06-16

**Authors:** Patrick D. M. C. Katoto, Sandra L. Mukasa, Mahmoud U. Sani, Kamilu M. Karaye, Irina Mbanze, Albertino Damasceno, Ana O. Mocumbi, Anastase Dzudie, Karen Sliwa, Friedrich Thienemann

**Affiliations:** 1 Cape Heart Institute and Department of Medicine, Faculty of Health Science, University of Cape Town, Cape Town, 792 South Africa; 2 Department of Medicine and Centre for Infectious Diseases, Faculty of Medicine and Health Sciences, Stellenbosch University, Cape Town, South Africa; 3 Faculty of Medicine and Centre for Tropical Diseases and Global Health, Catholic University of Bukavu, Bukavu, Democratic Republic of Congo; 4 Department of Medicine, Bayero University Kano & Aminu Kano Teaching Hospital, PMB 3011, Gwarzo Road, Kano, Kano Nigeria; 5 Faculty of Medicine, Eduardo Mondlane University, Dr Salvador Allende, Cp 257, Maputo, Mozambique; 6 Instituto Nacional de Saúde, Maputo, Mozambique; 7 Department of Internal Medicine, Douala General Hospital, PO Box 4856, Douala, Cameroon; 8 Department of Medicine, University Hospital Zurich, University of Zurich, Zurich, Switzerland

**Keywords:** Cardiovascular diseases, Infectious diseases, Respiratory tract diseases, Risk factors

## Abstract

In sub-Saharan Africa, little is known about pulmonary hypertension in left heart disease (PH-LHD). We used multivariate logistic and cox-hazard proportional regression models to examine factors associated with increased right ventricular systolic pressure (RVSP) and the effect of real-world HIV status scenarios on 6-month survival rate in the Pan African Pulmonary Hypertension Cohort (PAPUCO) study, a prospective cohort from four African countries. Exposure to biomass fuel smoke (aOR, 95%CI 3.07, 1.02–9.28), moderate to severe NYHA/FC III/IV (aOR, 95%CI 4.18, 1.01–17.38), and unknown HIV status (aOR, 95%CI 2.73, 0.96–7.73) predicted moderate to severe RVSP at the time of presentation. Six months later, HIV infection, moderate-to-severe NYHA/FC, and alcohol consumption were associated with decreased survival probabilities. Upon adjusting for HIV infection, it was observed that an incremental rise in RVSP (1 mmHg) and inter-ventricular septal thickness (1 mm) resulted in an 8% (aHR, 95%CI 1.08, 1.02–1.13) and 20% (aHR, 95%CI 1.2, 1.00–1.43) increase in the probability of mortality due to PH-LHD. In contrast, the risk of death from PH-LHD was reduced by 23% for each additional unit of BMI. (aHR, 95%CI 0.77, 0.59–1.00). In conclusion, the present study offers insights into the determinants that are notably linked to unfavorable survival outcomes in patients with pulmonary hypertension due to left heart disease. Certain factors identified in this study are readily evaluable and amenable to modification, even in settings with limited resources.

## Introduction

The Global Burden of Diseases has presented a spectacular decrease in overall HIV mortality [(from 1.95 in 2006 to 0.95 million deaths in 2017)] following antiretroviral therapy (ART) scale-up. This has consequently resulted in an increase in life-expectancy and consecutive increased HIV prevalence (36.8 million (CI 34.8–39.2) with the majority of HIV-infected persons residing in sub-Saharan Africa (SSA)^1^. Cardiovascular diseases (CVD) and metabolic complications in ageing patients living with HIV infection are consequently rising. They are multifactorial and in alongside to chronic inflammation, immune activation, endothelial dysfunction and haemostatic dysregulation; In addition, long-term ART toxicity and lifestyle are among hypothesized mechanisms leading for non-AIDS-defined conditions^2,3^. One of the most devastating sequelae during HIV infection is the increased risk of development of pulmonary hypertension (PH). PH is a devastating disease with high mortality rates. HIV increases the risk of PH by the factor 5000 when compared to HIV-uninfected adults. Unfortunately, PH often remains undiagnosed due to lack of cardiac workup in clinics in most regions of the world. A mean pulmonary artery pressure at rest of 25 mmHg or more, measured by right heart catheterization, defines it^4,5^. Although the exact mechanism remains unknown, HIV viral proteins and epigenetic triggers are implicated in its pathogenesis. ART alone without specific treatment for PH does not lead to improvement^2,6,7^.

Among the five groups of PH defined by the WHO based on pathophysiological, clinical, and therapeutic considerations; PH associated with left heart diseases (LHD) represents by far the most common form of PH accounting for 65–80% of PH cases. Patients with PH-LHD are often older, female, with a higher prevalence of cardiovascular co-morbidities. However, particular in developing regions where more than 80% of patients with PH are living, such a pathology is reported in younger patients than in developed countries^4,5,8^. This might be linked to additional risk factors of CVD in Africa such as rheumatic heart diseases, HIV infection and environmental risk factors (household air pollution from biomass fuel smoke and ambient air pollution)^9^ and might explain the particular profile of heart diseases in this region that are affecting persons at the age of high economic productivity. Equally, traditional risk factors for LHD (diabetes, hypertension, metabolic syndrome etc.) are increasing in Africa consecutive to the epidemiological transition. It is to be expected that LHD is rising in SSA^9,10^. PH in patients with LHD, is a maker of disease severity as patients experience more severe symptoms, worse tolerance to effort, higher hospitalization and mortality rates^5,11,12^. Moreover, PH-LHD is even more challenging for patients and doctors in the context of HIV infection especially in settings without integrated care.

Therefore, following the paucity of robust primary studies regarding clinical phenotype of PH-LHD and the effect of HIV infection on PH-LHD severity and death in Africa—and in the context that SSA carries the highest global burden of HIV infection—our study aimed to describe the socio-demographics, risk factors, clinical profile, management, short-term survival rate and the effect of HIV status in patients with PH-LHD in four African countries. To fill this critical gap, we used the Pan African Pulmonary Hypertension Cohort (PAPUCO) study, a contemporary registry on PH in Africa. We hypothesized that de novo PH-LHD is associated with younger age, higher severity, and lower 6 months survival rate among African people particularly in those living with HIV infection.

## Results

### Baseline sociodemographic, clinical, and biological data

**Figure 1:**
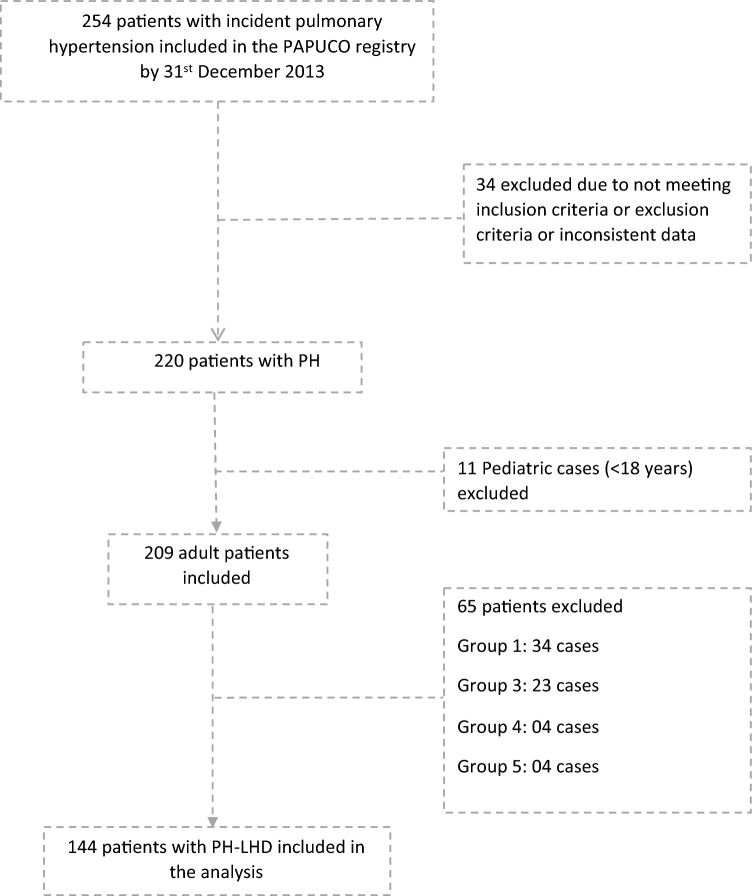
Flowchart showing the derivation of the pulmonary hypertension (PH) due to left heart disease cohort stratified by HIV status from the original PAPUCO all causes PH cohort. PH, pulmonary hypertension; PH-LHD, PH due to left heart disease.

Figure 1 depicts the flow diagram of the PAPUCO research. Sixty-nine percent (144/209) of all patients included in PAPUCO had PH-LHD.

Twenty percent (16/80) of patients who underwent HIV testing were tested positive for HIV (latest versus nadir mean (standard deviation-SD) CD4 count: 336.2 (204.1) vs 253.3 (212.9) cells per 10^6^/L). Furthermore, participants living with HIV were significantly younger (39 years), unemployed, were smokers and abused alcohol than those without HIV or with unknown HIV status. While the latter had a greater rate of self-reported hypertension and exposure to schistosomiasis, the former had a higher rate of previous tuberculosis (56.3%) (Table 1).

**Table 1:** Baseline sociodemographic, clinical, and biological features of 144 participants included in the Pan African Pulmonary Cohort presenting pulmonary hypertension due to left heart disease stratified by HIV status.

Variables	HIV status			All	P-value
	HIV-negative	HIV-positive	HIV-unknown		
ECG findings					
Sinusal rhythm/no	33 (58.9)	7 (46.7)	32 (56.1)	72 (56.3)	0.7
Sinus tachycardia/yes	9 (16.1)	5 (33.3)	20 (35.1)	34 (26.6)	0.05
Atrial fibrillation/yes	19 (33.9)	2 (13.3)	10 (17.5)	31 (24.2)	0.08
P pulmonal/yes	5 (8.9)	4 (26.7)	2 (3.5)	11 (8.6)	0.01
RV hypertrophy strain/yes	2 (3.6)	4 (26.7)	8 (14.0)	14 (10.9)	0.02
LV hypertrophy strain/yes	7 (12.5)	7 (46.7)	28 (49.1)	42 (32.8)	< 0.001
Chest X-ray findings					
Cardiomegaly/yes	39 (90.7)	9 (90.0)	37 (97.4)	85 (93.4)	0.3
Prominent pulmonary artery/yes	14 (32.6)	6 (60.0)	17 (44.7)	37 (40.7)	0.2
Echocardiography findings					
Heart rate (beats per minute)*	91.8 (17.6)	92.3 (26.5)	96.3 (18.3)	93.9 (19.2)	0.09
Aortic root size (mm)*	28.8 (5.3)	30.14 (5.3)	30.6 (5.1)	29.8 (5.2)	0.9
Left atrial size (mm)*	48.7 (11.2)	47.4 (7.1)	52.3 (9.9)	50.2 (10.4)	0.2
LV end diastolic (mm)*	53.8 (13.4)	61.8 (10.3)	59.6 (10.7)	57.6 (10.7)	0.2
LV end systolic (mm)*	40.7 (15.6)	50.7 (11.1)	47.3 (13.1)	44.7 (14.5)	0.2
IV septum systolic (mm)*	12.9 (3.7)	14.6 (4.9)	12.9 (3.7)	13.1 (3.8)	0.4
IV septum diastolic (mm)*	10.8 (3.0)	11.5 (2.8)	10.8 (2.9)	10.9 (2.9)	0.9
Fractional shortening (%)	25.3 (12.1)	16.7 (4.7)	21.2 (11.5)	22.2 (11.5)	0.009
EF calculated (%)	46.7 (19.4)	36.5 (17.8)	41.5 (18.2)	43.4 (18.9)	0.8
EF visual estimate (%)	42.7 (18.3)	40.1 (18.1)	40.8 (16.2)	41.6 (17.3)	0.7
Posterior wall diastolic diameter (mm)*	11.6 (3.1)	11.4 (2.9)	10.9 (2.7)	11.3 (4.6)	< 0.001
Posterior wall systolic diameter (mm)*	14.7 (7.1)	14.9 (3.3)	13.3 (3.3)	14.1 (5.4)	< 0.001
Mitral A-wave (m per second)*	39.9 (35.0)	18.9 (34.4)	44.9 (38.4)	38.6 (36.9)	0.8
Mitral E-wave (m per second)*	91.6 (53.5)	26.1 (32.4)	90.2 (48.6)	82.9 (53.0)	0.2
Deceleration time (ms)*	161.57 (76.9)	116.3 (55.1)	129.0 (41.8)	136.6 (47.8)	0.04
Regional wall motion abnormality/yes	5 (8.9)	2 (14.3)	9 (16.1)	16 (12.7)	0.5
Right atrial size					
Normal	8 (13.6)	1 (6.3)	4 (6.5)	13 (9.5)	0.04
Mildly enlarged	21 (35.6)	2 (12.5)	21 (33.9)	44 (32.1)	
Moderately enlarged	22 (37.3)	10 (62.5)	35 (56.5)	67 (48.9)	
Severely enlarged	8 (13.6)	3 (18.8)	2 (3.2)	13 (9.5)	
Right ventricle size					
Normal	15 (25.0)	2 (12.5)	6 (9.8)	23 (16.8)	0.06
Mildly enlarged	19 (31.7)	2 (12.5)	25 (40.9)	46 (33.6)	
Moderately enlarged	24 (40.0)	10 (62.5)	28 (45.9)	62 (45.3)	
Severely enlarged	2 (3.3)	2 (12.5)	2 (3.3)	6 (4.4)	
RVSP (mmHg)*	57.9 (16.1)	50.9 (6.8)	65.6 (17.6)	60.6 (16.7)	0.001
RVSP/type					
Mild	26 (41.9)	7 (43.8)	14 (21.9)	47 (33.1)	0.003
Moderate	16 (25.8)	8 (50.0)	16 (25.0)	40 (28.2)	
Severe	20 (32.3)	1 (6.3)	34 (53.1)	55 (38.7)	
TAPSE (mm)*	16.6 (6.8)	17.8 (16.7)	12.8 (4.4)	14.9 (7.7)	< 0.001
Aortic stenosis/yes	1 (1.67)	0 (0.0)	1 (1.7)	2 (1.5)	0.8
Mitral stenosis					
Mild	47 (81.0)	14 (87.5)	58 (96.7)	119 (88.8)	0.02
Moderate	2 (3.5)	1 (6.3)	1 (1.7)	4 (2.9)	
Severe	9 (15.5)	1 (6.3)	1 (1.7)	11 (8.2)	
Aortic regurgitation					
No or trace	35 (61.4)	12 (75.0)	38 (61.3)	85 (62.9)	0.08
Mid	20 (35.1)	2 (12.5)	13 (20.9)	35 (25.9)	
Moderate	2 (3.5)	2 (12.5)	7 (11.3)	11 (8.2)	
Severe	0 (0.0)	0 (0.0)	0 (0.0)	4 (6.5)	
Mitral regurgitation					
No or trace	18 (30.0)	4 (26.7)	8 (12.9)	30 (21.9)	0.001
Mid	23 (38.3)	3 (20.0)	15 (24.2)	41 (29.9)	
Moderate	17 (28.3)	5 (33.3)	20 (32.3)	41 (29.9)	
Severe	2 (3.3)	3 (20.0)	19 (30.7)	24 (17.5)	
Tricuspid regurgitation					
No or trace	3 (4.9)	1 (6.3)	2 (3.1)	6 (4.3)	0.5
Mid	7 (11.5)	1 (6.3)	5 (7.8)	13 (9.2)	
Moderate	41 (67.2)	9 (56.3)	38 (59.4)	88 (62.4)	
Severe	10 (16.4)	5 (31.3)	19 (29.7)	34 (24.1)	
Aetiology of PH-LHD					
Valvular disease	13 (20.3)	2 (12.5)	14 (21.9)	29 (20.1)	0.9
LV systolic dysfunction	35 (54.7)	11 (68.8)	35 (54.7)	81 (56.3)	
LV diastolic dysfunction	16 (25.0)	3 (18.8)	15 (23.4)	34 (23.6)	

Data are number (%) or mean ± SD.

BMI, body mass index; BP, blood pressure; COPD, Chronic obstructive pulmonary disease; CVD, cardiovascular disease; CRP, C reactive protein; FU, follow up; HIV, human immunodeficiency syndrome; HR, heart rate, JVP, jugular venous pressure; L(R)VH, Left(right) ventricular hypertrophy; PH, pulmonary hypertension; RVSP, right ventricular systolic pressure; USD, US dollar; WHO, World Health Organization; 6MWT, 6-min’ walk test., bpm: breaths per minute.

Data are expressed as n (%), * indicates continuous values, expressed as mean (standard deviation).

During clinical evaluation, HIV-infected individuals had a relatively low BMI (22.6 vs 25.0 kg/m^2^) but a significantly higher baseline respiratory rate at rest (28 vs. about 25 bpm) than those without HIV or with unknown HIV status. Aside from glycemia, which was significantly higher in people with unknown HIV status (vs. those who tested positive or negative for HIV), other biomarkers such as serum creatinine, alanine aminotransferase, and triglyceride levels were significantly altered in HIV-infected participants compared to their counterparts.

### Availability of PH-targeted treatment

Only 9% of those who took part in the study received PH-targeted medication (calcium channel blockers and/or phosphodiesterase inhibitors). Participants with HIV infection received more loop diuretics per dosage (110.8 mg/dose) than those without HIV infection (73.7 mg/dose) or with uncertain HIV status (65.1 mg/dose). Only 5.6% of those polled had access to home oxygen (Table 1).

### Baseline electrocardiogram, chest x-ray, and echocardiography findings

On ECG evaluation, more than half of the individuals (56%) did not have a sinus rhythm, with atrial fibrillation being more common among those with unknown HIV status and those without HIV infection, respectively. The latter group also had a higher prevalence of left ventricular hypertrophy with strain than the rest. P pulmonale and ventricular strains, on the other hand, were substantially more common in HIV-infected people than in the other groups (Table 2). Cardiomegaly was found on x-ray in all groups. When compared to other groups, echocardiographic data revealed that more people with HIV had shortened deceleration time and a moderately to substantially enlarged right atrial size. Overall, RVSP was 60.6 (16.7) mmHg on average (standard deviation). According to the RVSP cut-off, most participants with unclear HIV status (53%) had severe PH, the majority of those with HIV infection (half) had moderate PH, and the majority of those without HIV infection (41.9%) had mild PH. While individuals living with HIV had a higher prevalence of mild mitral stenosis and regurgitation than the other groups, those without HIV and those with unknown HIV infection had a larger proportion of mitral stenosis and regurgitation.

**Table 2:** Baseline electrocardiographic, chest x-ray and echocardiographic findings of 144 participants included in the Pan African Pulmonary Cohort presenting pulmonary hypertension due to left heart disease stratified by HIV status.

Variables	HIV status			All	P-value
	HIV-negative	HIV-positive	HIV-unknown		
ECG findings					
Sinusal rhythm/no	33 (58.9)	7 (46.7)	32 (56.1)	72 (56.3)	0.7
Sinus tachycardia/yes	9 (16.1)	5 (33.3)	20 (35.1)	34 (26.6)	0.05
Atrial fibrillation/yes	19 (33.9)	2 (13.3)	10 (17.5)	31 (24.2)	0.08
P pulmonal/yes	5 (8.9)	4 (26.7)	2 (3.5)	11 (8.6)	0.01
RV hypertrophy strain/yes	2 (3.6)	4 (26.7)	8 (14.0)	14 (10.9)	0.02
LV hypertrophy strain/yes	7 (12.5)	7 (46.7)	28 (49.1)	42 (32.8)	< 0.001
Chest X-ray findings					
Cardiomegaly/yes	39 (90.7)	9 (90.0)	37 (97.4)	85 (93.4)	0.3
Prominent pulmonary artery/yes	14 (32.6)	6 (60.0)	17 (44.7)	37 (40.7)	0.2
Echocardiography findings					
Heart rate (beats per minute)*	91.8 (17.6)	92.3 (26.5)	96.3 (18.3)	93.9 (19.2)	0.09
Aortic root size (mm)*	28.8 (5.3)	30.14 (5.3)	30.6 (5.1)	29.8 (5.2)	0.9
Left atrial size (mm)*	48.7 (11.2)	47.4 (7.1)	52.3 (9.9)	50.2 (10.4)	0.2
LV end diastolic (mm)*	53.8 (13.4)	61.8 (10.3)	59.6 (10.7)	57.6 (10.7)	0.2
LV end systolic (mm)*	40.7 (15.6)	50.7 (11.1)	47.3 (13.1)	44.7 (14.5)	0.2
IV septum systolic (mm)*	12.9 (3.7)	14.6 (4.9)	12.9 (3.7)	13.1 (3.8)	0.4
IV septum diastolic (mm)*	10.8 (3.0)	11.5 (2.8)	10.8 (2.9)	10.9 (2.9)	0.9
Fractional shortening (%)	25.3 (12.1)	16.7 (4.7)	21.2 (11.5)	22.2 (11.5)	0.009
EF calculated (%)	46.7 (19.4)	36.5 (17.8)	41.5 (18.2)	43.4 (18.9)	0.8
EF visual estimate (%)	42.7 (18.3)	40.1 (18.1)	40.8 (16.2)	41.6 (17.3)	0.7
Posterior wall diastolic diameter (mm)*	11.6 (3.1)	11.4 (2.9)	10.9 (2.7)	11.3 (4.6)	< 0.001
Posterior wall systolic diameter (mm)*	14.7 (7.1)	14.9 (3.3)	13.3 (3.3)	14.1 (5.4)	< 0.001
Mitral A-wave (m per second)*	39.9 (35.0)	18.9 (34.4)	44.9 (38.4)	38.6 (36.9)	0.8
Mitral E-wave (m per second)*	91.6 (53.5)	26.1 (32.4)	90.2 (48.6)	82.9 (53.0)	0.2
Deceleration time (ms)*	161.57 (76.9)	116.3 (55.1)	129.0 (41.8)	136.6 (47.8)	0.04
Regional wall motion abnormality/yes	5 (8.9)	2 (14.3)	9 (16.1)	16 (12.7)	0.5
Right atrial size					
Normal	8 (13.6)	1 (6.3)	4 (6.5)	13 (9.5)	0.04
Mildly enlarged	21 (35.6)	2 (12.5)	21 (33.9)	44 (32.1)	
Moderately enlarged	22 (37.3)	10 (62.5)	35 (56.5)	67 (48.9)	
Severely enlarged	8 (13.6)	3 (18.8)	2 (3.2)	13 (9.5)	
Right ventricle size					
Normal	15 (25.0)	2 (12.5)	6 (9.8)	23 (16.8)	0.06
Mildly enlarged	19 (31.7)	2 (12.5)	25 (40.9)	46 (33.6)	
Moderately enlarged	24 (40.0)	10 (62.5)	28 (45.9)	62 (45.3)	
Severely enlarged	2 (3.3)	2 (12.5)	2 (3.3)	6 (4.4)	
RVSP (mmHg)*	57.9 (16.1)	50.9 (6.8)	65.6 (17.6)	60.6 (16.7)	0.001
RVSP/type					
Mild	26 (41.9)	7 (43.8)	14 (21.9)	47 (33.1)	0.003
Moderate	16 (25.8)	8 (50.0)	16 (25.0)	40 (28.2)	
Severe	20 (32.3)	1 (6.3)	34 (53.1)	55 (38.7)	
TAPSE (mm)*	16.6 (6.8)	17.8 (16.7)	12.8 (4.4)	14.9 (7.7)	< 0.001
Aortic stenosis/yes	1 (1.67)	0 (0.0)	1 (1.7)	2 (1.5)	0.8
Mitral stenosis					
Mild	47 (81.0)	14 (87.5)	58 (96.7)	119 (88.8)	0.02
Moderate	2 (3.5)	1 (6.3)	1 (1.7)	4 (2.9)	
Severe	9 (15.5)	1 (6.3)	1 (1.7)	11 (8.2)	
Aortic regurgitation					
No or trace	35 (61.4)	12 (75.0)	38 (61.3)	85 (62.9)	0.08
Mid	20 (35.1)	2 (12.5)	13 (20.9)	35 (25.9)	
Moderate	2 (3.5)	2 (12.5)	7 (11.3)	11 (8.2)	
Severe	0 (0.0)	0 (0.0)	0 (0.0)	4 (6.5)	
Mitral regurgitation					
No or trace	18 (30.0)	4 (26.7)	8 (12.9)	30 (21.9)	0.001
Mid	23 (38.3)	3 (20.0)	15 (24.2)	41 (29.9)	
Moderate	17 (28.3)	5 (33.3)	20 (32.3)	41 (29.9)	
Severe	2 (3.3)	3 (20.0)	19 (30.7)	24 (17.5)	
Tricuspid regurgitation					
No or trace	3 (4.9)	1 (6.3)	2 (3.1)	6 (4.3)	0.5
Mid	7 (11.5)	1 (6.3)	5 (7.8)	13 (9.2)	
Moderate	41 (67.2)	9 (56.3)	38 (59.4)	88 (62.4)	
Severe	10 (16.4)	5 (31.3)	19 (29.7)	34 (24.1)	
Aetiology of PH-LHD					
Valvular disease	13 (20.3)	2 (12.5)	14 (21.9)	29 (20.1)	0.9
LV systolic dysfunction	35 (54.7)	11 (68.8)	35 (54.7)	81 (56.3)	
LV diastolic dysfunction	16 (25.0)	3 (18.8)	15 (23.4)	34 (23.6)	

Data are expressed as n (%), * indicates continuous values, expressed as mean (standard deviation).

LV, left ventricular, RV: right ventricular, IV: intraventricular, RVSP: right ventricular systolic pressure, TAPSE: tricuspid annular plane systolic excursion, EF: ejection fraction.

### Predictors of raised RVSP at baseline in PH-LHD patients

**Figure 2:**
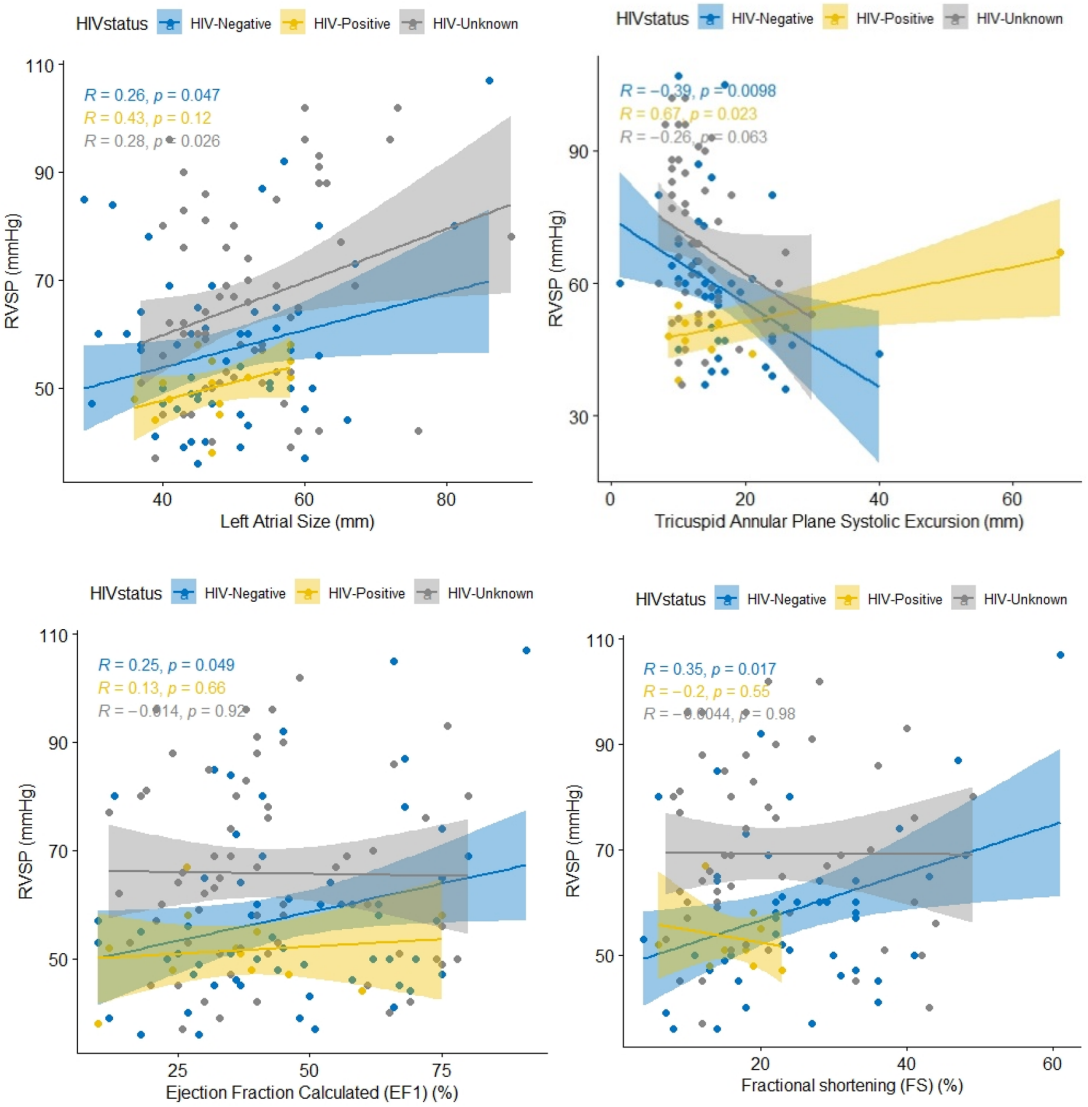
Correlation between right ventricular systolic pressure (RVSP) and left atrial size, tricuspid annular plane systolic excursion, ejection calculated and fractional shortening by HIV status among patients with pulmonary hypertension due to left heart diseases in the pan African pulmonary hypertension cohort (PAPUCO).

RVSP showed a low positive but statistically significant correlation with left atrial size in people without HIV (r = 0.26) and those with unknown HIV status (r = 0.28). It also showed a moderate positive and statistically significant correlation (r = 0.67) among people living with HIV, but a low negative and statistically significant correlation (r = − 0.39) and a low negative and statistically significant correlation (r = − 0.26) among participants without HIV (r = − 0.26) with TAPSE. Furthermore, among non-HIV-infected patients, RSVP exhibited a low but statistically significant positive correlation with estimated ejection fraction (r = 0.25) and fraction shortening (r = 0.35) (Fig. 2).

After controlling for other variables in the multivariable logistic regression model, exposure to biomass fuel smoke used for domestic energy (cooking, heating, or lighting) (aOR, 95%CI 3.07, 1.02 to 9.28), having moderate to severe dyspnoea/NYHA FC III/IV (aOR, 95%CI 4.18, 1.01 to 17.38), and having unknown HIV status were independently associated with moderate to severe RVSP at admission (Table 3).

**Table 3:** Multivariate logistic and cox-proportional hazards regressions of predictors associated with raised RVSP at baseline and death at 6 months of follow-up, respectively.

Variables	Baseline moderate to severe RVSP^a^		Mortality rate at 6 months			
	aOR, 95%CI	P value	HIV-unadjusted^b^		HIV-adjusted^c^	
			aHR, 95%CI	P value	aHR, 95%CI	P value
Use of biomass fuel	3.07, 1.02–9.28	0.046				
Unknown HIV status	2.73, 0.96–7.73	0.07				
NYHA FC III/IV	4.18, 1.01–17.38	0.049	4.2, 0.96–18.47	0.058	5.03, 1.53–16.51	0.008
RVSP (mmHg)			1.07, 1.03–1.11	0.001	1.08, 1.02–1.13	0.004
IVSS			1.25, 1.07–1.46	0.005	1.2, 1.00–1.43	0.051
BMI (kg/m^2^)			0.75, 0.8–0.98	0.034	0.77, 0.59–1.00	0.05
Alcohol abuse			4.77, 0.60–37.97	0.14	7.14, 1.04–49.07	0.046

RVSP: right ventricular systolic pressure, NYHA/FC: New York Heart Association Functional Classification, BMI: Body Mass Index, IVSS: interventricular septum systolic, TAPSE: tricuspid annular plane systolic excursion.

^a^Model adjusted for sex, age, occupation type, diabetes, hypertension, exposure to schistosomiasis, alcohol abuse, rheumatic diseases, peripheral oedema, and 6 min walking distance.

^b^Model adjusted for age, sex, smoking habit, left atrial size, TAPSE, pulse at rest, hypercholesterolemia, alcohol abuse and ejection fraction calculated (EF).

^c^Model adjusted for as for ^(b)^ plus HIV status.

### Predictors of 6-month mortality in PH-LHD patients

**Figure 3:**
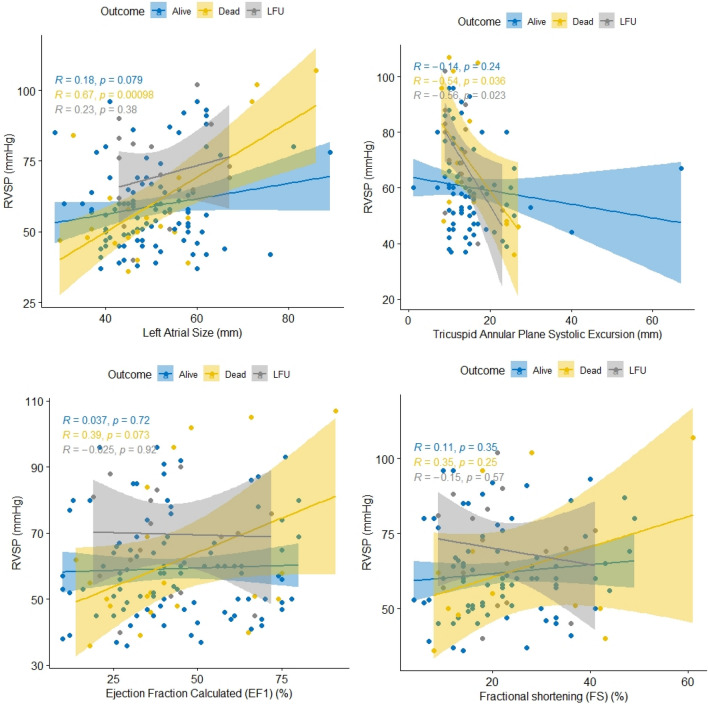
Correlation between right ventricular systolic pressure (RVSP) and left atrial size, tricuspid annular plane systolic excursion, ejection calculated and fractional shortening by 6-month survival outcome among patients with pulmonary hypertension due to left heart diseases in the pan African pulmonary hypertension cohort (PAPUCO).

The death rate after 6 months of follow-up was 15%. We found a moderate positive and statistically significant link between RVSP and left atrial size (r = 0.67), as well as a moderate negative correlation between RVSP and TAPSE (r = − 0.54) in deceased individuals. There was no statistically significant correlation between RVSP and estimated ejection fraction or fraction shortening when patients were categorized based on survival outcomes (Fig. 3).

**Figure 4:**
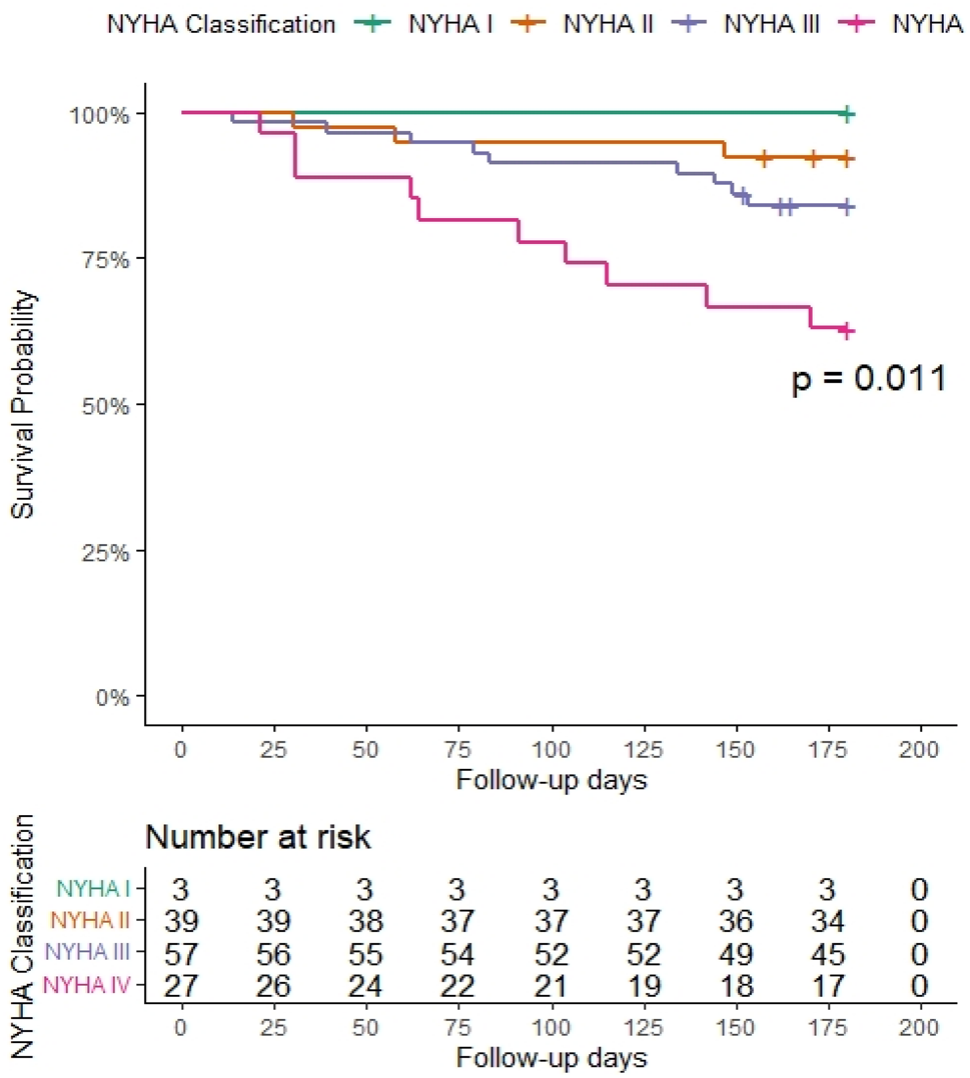
Kaplan-Meilleur survival curve after 6-month follow-up period of patients with pulmonary hypertension due to left heart diseases according to baseline New York functional class (NYHA) categorization in the pan African pulmonary hypertension cohort (Cohort).

Figure 4 displays the percentages of PH-LHD patients who died at various points throughout the 6-month follow-up period (180 days), based on their initial NYHA classification. The likelihood of survival frequently follows a monotonic trend. Rates are comparable across all NYHA categories until about 25 days. Deaths for the NYHA IV group skyrocket at this time and continue to climb for the rest of the period. After 75 days, participants in the NYHA III group died at a somewhat higher and consistent rate than those in the NYHA II group, with a substantial difference continuing at least until 150 days.

**Figure 5:**
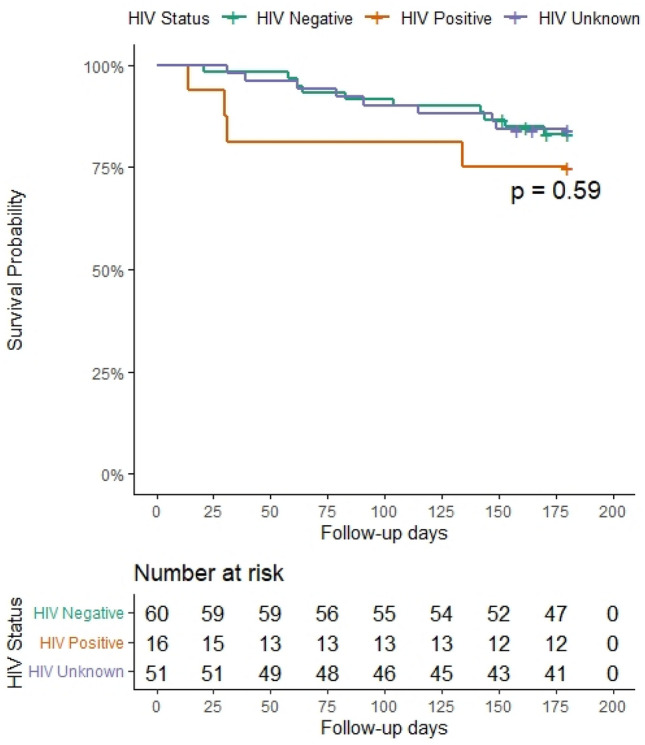
Kaplan–Meilleur survival curve after 6-month follow-up period of patients with pulmonary hypertension due to left heart diseases according to HIV status in the pan African pulmonary hypertension cohort (Cohort).

Furthermore, after 6 months of follow-up, the survival probabilities for the PH-LHD among individuals living with HIV were lower than those living without HIV or with unknown HIV status. However, the change was not statistically significant (Fig. 5).

To investigate the influence of HIV infection and other predisposing variables on PH-LHD-related mortality, two Cox proportional hazards regression models (unadjusted and adjusted for HIV infection) were created. After accounting for HIV infection, we found that for each incremental increase in one unit of RVSP and interventricular septal thickness in systole (IVSS, the risk of dying from PH-LHD rose by 8% (adjusted hazard ratio [aHR], 95% CI 1.08, 1.02–1.13) and 20% (aHR, 95% CI 1.2, 1.00–1.43), respectively. However, for each additional unit of BMI, the risk of dying from PH-LHD decreased by 23%. (aHR, 95% confidence interval: 0.77, 0.59–1.00). Furthermore, participants with moderate to severe NYHA and a history of alcohol abuse were five times (aHR, 95% CI 5.03, 1.53–16.51) and seven times (aHR, 95% CI 7.14, 1.04–49.07) more likely to die from PH-LHD than those with mild NYHA and no history of alcohol abuse at baseline, respectively. In the unadjusted model for HIV infection, a similar trend was seen, however the risk associated with NYHA was borderline, and the risk associated with a history of alcohol usage was simply not statistically significant (Table 3).

## Discussion

In this prospective longitudinal study, we assessed socio-demographics, risk factors, clinical profile, and short-term survival rate of pulmonary hypertension due to left heart disease in relation to HIV infection in four African countries. We found that participants who were HIV-infected were significantly younger, unemployed, smokers and drank alcohol than those who were not HIV-infected or had an unknown HIV status. We also found that participants who were exposed to biomass fuel smoke used for domestic energy, had moderate to severe dyspnoea/NYHA FC III/IV, or had an unknown HIV status were more likely to have moderate to severe RVSP at admission. After 6 months of follow-up, we found that the survival probability for PH-LHD among HIV-infected individuals was lower than the survival probability for those living without HIV infection or with unknown HIV status. Furthermore, after controlling for HIV infection, we found that history of alcohol consumption, NYHA FC II/IV, BMI, RVSP, and IVSS were independent predictors of mortality associated with PH-LHD. Survival probability has followed a monotone pattern according to patients' initial NYHA FC, with those classified as NYHA IV demonstrating a considerably lower survival probability over time.

Our data support the notion that the pandemics of PH and left-sided heart disease are no longer isolated to high-income countries, but have spread to various African regions^5^. In our study, like in many settings with varying resources, transthoracic doppler echocardiography-measured right ventricular systolic pressure has been utilized as a surrogate for identifying PH. Although the precise hemodynamic threshold at which RVSP is inextricably connected with mortality is controversial, we found that after correcting for other factors in the model, the likelihood of dying from PH-LHD rose by 8% for each 1 mmHg incremental of RVSP. Similar to our finding, after adjusting for confounding factors such as the degree of LVSD and the presence of chronic obstructive pulmonary disease, a 5 mmHg increase in RVSP was associated with a corresponding 6% increase in mortality in the largest population study in Scotland^13^ (n = 1612 patients, mean follow-up was 2.8 ± 2.5). These findings support the notion that among individuals with LVSD, even a minor rise in RVSP was associated with a higher risk of death.

Participants in our cohort who were living with HIV were significantly younger (39 years), less likely to be employed and more likely to have a history of substance abuse than those who were not HIV-infected or had an unknown HIV status. This profile is quite atypical as more than 80% of heart failure (HF) patients in Europe and the United States are 65 or older^5^ and this, regardless of either HF has a reduced or a preserved ejection fraction. Furthermore, if LV ejection fraction is affected, patients are more likely to present with a history of cardiovascular co-morbidities and trait of metabolic syndrome^4^. When considering the impact of HIV infection on LHD presentation, in a meta-analysis^14^ of 125,382 PLWHIV involving 12,665 cases of cardiac dysfunction; LV dysfunction as well as PH were common and studies with a greater proportion of individuals with AIDS or a lower proportion taking ART indicated a higher frequency of LVSD. This is congruent with our findings, since LVSD was present in more than half of our PH-LHD patients. Furthermore, we found that left atrial (LA) size was associated with death in our patients, with a modest correlation in PLWHIV. However, LA remodelling leads to the development of PH-LHD, as evidenced by increased LA size, poorer LA contractility, and interstitial fibrosis leading to LA stiffness and reduced compliance. If the changes in LA pressure continue, they may cause structural anomalies in the pulmonary vasculature, such as intimal fibrosis and medial hypertrophy, resulting in diminished vasodilator response, pulmonary vasoconstriction, and higher PVR, and eventually mortality in PH-LHD patients^15^.

We observed that a baseline increased in IVSS predicted mortality in individuals with PH-LHD. Several investigations have found that IVS has prognostic importance. A study that looked at the relationship between left ventricular structure and the development of hypertension in a young healthy population (n = 500 air force men with a mean age of 20.5 ± 3.3 (range, 17–40) years and a baseline BP of 125 ± 13/74 ± 8 mmHg.) found that the likelihood that systolic BP would be higher than the median during follow-up was twice as high in those with an IVS thickness greater than the median. As a result, this simple measure can assist in predicting a PH-LHD patient who is at risk for SBP, which is also a risk factor for left heart disease^16^. Furthermore, IVS end-systolic flattening has been shown to be a sensitive indicator for RV systolic hypertension. According to the Sub-Saharan acute heart failure study, which was conducted in nine African countries, the clinical significance will need to be assessed in a large cohort because the high prevalence of PH-LHD in LMICs is expected due to a higher prevalence of uncontrolled hypertension, which is also a major cause of heart failure^17,18^.

It is clear that PH-LHD in SSA has the same conventional risk factors for cardiovascular disease as in developing nations, but the underlying burden of chronic infectious illnesses and poverty may have an influence on the disease's natural history. For example, after 6 months of follow-up, the survival probability for the PH-LHD among individuals living with HIV in our cohort was lower than the survival probability among those living without HIV. As a result, our findings support the need for a screening of at-risk individuals as well as the treatment of subclinical cardiac dysfunction found utilizing imaging techniques^14^. We also found that clinical indicators that doctors may easily identify in settings with limited resources, such as NYHA FC III or IV and lower BMI, predicted poor survival in our population. PH, a common consequence of LHDs, can aggravate any left heart issue and occurs commonly as a 'symptom' of the underlying illness and is generally associated to disease severity. In left-sided valvular disorders, for example, the incidence of PH increases with the severity of the defect and the intensity of the symptoms. The frequency of PH increases with the advancement of FC impairment in individuals with chronic HF. When PH-LHD is present, it causes more severe symptoms and worse exercise tolerance, and it has a detrimental influence on outcome^4^. As a result, severe NYHA is a strong and easily accessible predictor of poor PH-LHD survival and, in some cases, appears to be a superior predictor when compared to the prognostic ability of sophisticated hemodynamic factors such as mean right atrial pressure, transpulmonary pressure gradient, pulmonary vascular resistance, and diastolic pulmonary vascular pressure gradient in patients with PH-LHD^11,19^.

Current data supports the existence of the obesity paradox in HF, in which patients with overweight or class 1 obesity have better clinical outcomes than patients with normal weight and equal degrees of HF, and a similar advantage on CVD outcome has been documented with BMI and WC^20^. In terms of PH, obese individuals with precapillary PH had a lower death rate than nonobese peers^21^. Comparable to our data, a retrospective cohort analysis of 110,495 veterans diagnosed with PH in the United States found that greater BMI was associated with lower mortality in a J-shaped pattern, with underweight and normal weight veterans having the highest risk regardless of the type of PH^22^. It has been proposed that lower BMI is associated with less energy stored in fat and lean mass (which may worsen known maladaptive increases in glycolytic metabolism in the failing heart); adipose tissue can exert beneficial endocrine and physiologic effects as well as paracrine effects in the perivascular space (reducing vasoconstriction, inflammation, and proliferation in systemic vessels); and subjects with obesity and HF can have higher cardiac output and systemic BP (may allow more liberal use of cardio- beneficial medications)^22–26^. While this beneficial feature can be lost in obesity with diabetes mellitus due to a proinflammatory adipocyte phenotype^27^, maintaining lean mass in the care of PH patients may be of more relevance in a community with a higher number of persons living with HIV and tuberculosis.

Exposure to biomass fuel smoke used for domestic energy (cooking, heating, or lighting), or household air pollution, was another substantial and modifiable risk factor independently linked with moderate to severe RVSP at admission (HAP). Exposure to HAP, such as wood smoke particulate matter (PM), causes increased oxidative stress and the generation of reactive oxygen species, as well as increased secretion of proinflammatory cytokines such as tumour necrosis factor alpha and changes in endothelin receptor expression^28–30^. Because HAP exposure and other factors such as alcohol misuse have been shown to have a negative impact on cardiovascular function, advocates for clean energy and increased clinician emphasis on eliminating alcohol use^9^ among people living with HIV may help improve the survival rate of those with co-existing heart disease in general and those with PH.

A major strength of our study is that we demonstrated that patients with PH-LHD had many risk factors, and as a result, we accounted for baseline differences and the effect of relevant variables such as HIV status by fitting several models for multivariate analysis. As a result, we have reported basic clinical variables as well as imaging lesions that may be used to manage PH in a place with limited resources. For example, because most of the studies described above were retrospective, they were hampered by a lack of NYHA functional class information that was not captured in the database. Our study has the advantage of including this factor in the final model because it represents an important and low-cost diagnostic and prognostic marker in patients with PH-LHD, as it predicted increased RVSP and those classified as NYHA IV exhibiting a low survival probability over time, with a significant difference beginning after 25 days and lasting until the end of the follow-up period. Our data further support the notion that an increased RVSP due to LVSD is an alarming indicator of PH diagnosis irrespective of chronic respiratory illnesses and the degree of LVSD, and that it is a significant measure of survival regardless of HIV status. While we recognize the distinction between transthoracic and its gold standard right catheterization in the diagnosis and management of PH, we also believe that, due to the scarcity of qualified human resources and equipment across SSA, as well as the significant risk factors for CVD and PH in young people living in SSA, a simple algorithm involving RVSP can provide data on PH where it is most needed. Further, the youngest age of our participants (mean age of our participants 53.7 ± 17.2 vs 75.2 ± 10.9), the highest mean RVSP (mean RVSP: 60.6 ± 16.7 vs 44.9 ± 13.1 mmHg), and a significant proportion of people living with HIV infection (20% of participants with valid test) and with a lower CD4 count (latest vs nadir mean (SD) CD4 count: 336.2 (204.1) vs 253.3 (212.9) cells per 10^6^/L) may restrict the generalizability of our findings, but it is also a call to action to adequately document PH-LHD in Africa.

## Conclusion

Individuals with HIV infection exhibited decreased PH-LHD survival likelihood at 6 months of follow-up, and RVSP and BMI were significant predictors of PH-LHD mortality, even after controlling for HIV infection. Patients with PH-LHD who had moderate to severe NYHA symptoms at the onset of the study and a history of alcohol abuse had a higher probability of death. Compared to patients with mild NYHA symptoms and no history of alcohol misuse at the start of the study, the former group had a fivefold mortality risk increase, while the latter had a sevenfold increase. To enhance the management of cardiovascular disease among the young population in Sub-Saharan Africa, it is imperative to give more consideration to the predictors of survival and modifiable predictors that increase RVSP at baseline. These predictors include exposure to air pollution and systematic screening for HIV status. The successful implementation of this endeavor necessitates a coordinated effort and partnership among governmental bodies, international organizations, and healthcare practitioners. Subsequent studies should aim to develop an end-user algorithm that facilitates the prompt detection and management of PH-LHD, specifically in individuals who are living with HIV.

## Methods

### Ethics consideration

All participating centres received ethical approval from their countries; Cameroon (comite national d’ethique de la recherche pour la sante humaine, No2013/11/363/L/CNERSH/SP), Mozambique (Ministerio da saude, comite nacional de bioetica a saude: IRB00002657), Nigeria (Lagos university teaching hospital, HREC, ADM/DCST/HREC/VOL.XVI/79) and South Africa (University of Cape Town, faculty of health science, HREC: FWA0000163; IRB00001938). The study adheres to the Helsinki Declaration's standards^31^. Prior to study enrolment, all individuals provided written informed consent, and HIV testing was conducted in accordance with national guidelines with consent of the participant.

### Study design, population and setting

The broad design, aims and specific methods underlying the PAPUCO study, the largest contemporary cohort study of PH in Africa, have been described in detail previously^31^ and registered at ClinicalTrials.gov (NCT02265887).Wherever possible, the registry adheres to the STROBE guidelines for reporting observational outcomes^32^. The PAPUCO research group was established in 2011 with the aim of establishing a prospective cohort study of de novo PH cases representative of the wider African population. Consequently, nine specialist care referral centres in Cameroon, Mozambique, Nigeria, and South Africa contributed to the registry. Each site recruited consecutive patients on the following basis: newly diagnosed with PH based on standardized clinical and echocardiography criteria, the capacity to return for 6-month follow-up if alive, aged ≥ 18-years (except for paediatric centres in Mozambique, Cameroon, and Nigeria) and provide written informed consent to participate. Centre eligibility included: availability of echocardiography and training in assessing right heart function, experience in diagnosing PH according to World Health Organization (WHO) classification, experience in clinical management of patients with right heart failure (RHF) and resources to review patients at 6-month follow-up. We have conducted a secondary data-analysis on the PAPUCO dataset which has data on HIV-positive and negative patients.

### Measuring PH in setting with variables resources

A diagnostic algorithm to detect PH and PH-LHD in resource-constraint settings without access to right heart catheterization had been established following the guidelines for the diagnosis of PH by the European Society of Cardiology (ESC) and European Respiratory Society (ERS)^4^. On this basis, PH was diagnosed by specialist cardiologists and defined as documented elevated right ventricular systolic pressure (RVSP) ≥ 35 mm Hg on transthoracic echocardiography in the absence of pulmonary stenosis and acute RHF; usually accompanied by dyspnoea, fatigue, peripheral oedema and other cardiovascular symptoms, ECG and chest X ray changes in keeping with PH. Additional investigations such as computed tomography, ventilation/perfusion scans or right heart catheterizations were performed at the discretion of the treating physician if available.

### Independent variables

The following data were documented for each participant: socio-demographic and ethnic profile, antecedent risk profile (including environmental exposures and cardiovascular risk factors), medical history, relevant clinical findings, prescribed treatment and management, all major cardiovascular diagnoses according to International Classification of Diseases (ICD) 10 coding and up to five non-cardiovascular diagnoses according to ICD-10 coding^4,33^. Clinical assessment included symptoms scoring, a full clinical examination, physical and clinical status. Functional tests included WHO Functional Class (FC), 6-min walk test (6MWT), and Karnofsky Performance Score. Technical procedures included echocardiography, chest X-ray, and 12-lead ECG.

### Dependent variables

Patient outcomes, including hospitalization and death during planned 6-month follow-up were also prospectively collected. A verbal autopsy was performed to record survival at the end of the study and survival data for the 144 participants with PH-LHD were retrieved from the registry using a Microsoft Excel sheet.

### Data management and statistical analysis

At least two investigators assessed each case to ensure that the data was comprehensive and genuine. All study data were collected using electronic case report forms (web-based platform) and kept in a secure central database. To investigate the influence of HIV infection in our practice as it occurs in the real world, we classify participants into those with known HIV status (i.e., following testing or being on antiretroviral therapy) and those with unknown HIV status (i.e., those who are not known for HIV or who refused to undertake HIV testing but enrolled in PAPUCO cohort). We employed visual assessment of boxplots and the D'Agostino-Pearson omnibus normality test to evaluate dependent variables for normality. To begin, we examined the sample using descriptive statistics. Using one-way ANOVA, chi-squared, or Fisher's exact tests, baseline data were compared between HIV status groups (negative, positive, and unknown). Using a Spearman correlation, we next examined the relationship between the RVSP in mmHg and left atrial size (mm), TAPSE (mm), Ejection fraction computed (percent), and fraction shortening (percent) by HIV infection status as well as survival status (alive, deceased, or lost to follow up). To explore factors associated with moderate to severe RVSP at admission, a multivariate logistic regression was performed. RVSP values were evaluated as a binary result (mild = 0, moderate to severe = 1). The model contained predictors that were found to have a relationship with moderate to severe RVSP at admission at a 0.2 level of significance. To arrive at a final model, backward selection based on likelihood ratios was performed. For odds ratios, the 95% CI was provided. To compare groups in terms of survival, we generated Kaplan–Meier survival estimates and applied the log-rank test. The relationship between risk variables and time to death was investigated using univariable and multivariable Cox proportional hazards regression models. The Nelson-Aalen cumulative hazard function and the Schoenfeld residuals test were used to evaluate the proportional hazard assumption. Two multivariable models were built, one for HIV-unadjusted and one for HIV-adjusted mortality, utilizing factors known from prior work to be highly linked with outcome and not collinear. We compared several regression models using the Akaike information criteria and chose the most parsimonious model. To summarize the magnitude of the association, we utilized the hazards ratio and the associated 95%CI interval. All p-values were two-sided, with p 0.05 indicating the threshold of significance. For data cleaning and analysis, R (The R Foundation for Statistical Computing, Vienna, Austria) V4.0.5 and GraphPad prism (v8.01) tools were used.

### Institutional review board statement

All participating centres received ethical approval from their local ethics committees. The study conformed to the principles of the 1975 Declaration of Helsinki.

### Informed consent statement

Written informed consent was obtained from all participants prior to study enrolment and HIV testing was performed, according to national guidelines, with consent.

## Data Availability

Data are available to the corresponding author upon a reasonable request.
